# Novel Mutation in the Acetohydroxyacid Synthase (AHAS), Gene Confers Imidazolinone Resistance in Chickpea *Cicer arietinum* L. Plants

**DOI:** 10.3390/plants10122791

**Published:** 2021-12-16

**Authors:** Shmuel Galili, Joseph Hershenhorn, Marvin Edelman, Vladimir Sobolev, Evgeny Smirnov, Orit Amir-Segev, Aharon Bellalou, Evgenia Dor

**Affiliations:** 1 Institute of Plant Sciences, The Volcani Center, Agricultural Research Organization, P.O. Box 15159, HaMaccabim Road 68, Rishon LeZion 7528809, Israel; oritas@volcani.agri.gov.il (O.A.-S.); aharonb@volcani.agri.gov.il (A.B.); 2 Newe Ya’ar Research Center, Agricultural Research Organization, P.O. Box 1021, Ramat Yishay 3009503, Israel; hershenj@gmail.com (J.H.); evgeny@volcani.agri.gov.il (E.S.); 3 Department of Plant and Environmental Sciences, Weizmann Institute of Science, Rehovot 76100, Israel; marvin.edelman@weizmann.ac.il (M.E.); vovsobo@gmail.com (V.S.)

**Keywords:** imidazolinone herbicide, herbicide resistance, acetohydroxyacid synthase (AHAS), chickpea, *Cicer arietinum* L., mutagenesis

## Abstract

Chickpea (*Cicer arietinum* L.) is an important crop in crop-rotation management in Israel. Imidazolinone herbicides have a wide spectrum of weed control, but chickpea plants are sensitive to acetohydroxyacid synthase (AHAS; also known as acetolactate synthase [ALS]) inhibitors. Using the chemical mutagen ethyl methanesulfonate (EMS), we developed a chickpea line (M2033) that is resistant to imidazolinone herbicides. A point mutation was detected in one of the two genes encoding the AHAS catalytic subunit of M2033. The transition of threonine to isoleucine at position 192 (203 according to *Arabidopsis*) conferred resistance of M2033 to imidazolinones, but not to other groups of AHAS inhibitors. The role of this substitution in the resistance of line M2033 was proven by genetic transformation of tobacco plants. This resistance showed a single-gene semidominant inheritance pattern. Conclusion: A novel mutation, T192I (T203I according to *Arabidopsis*), providing resistance to IMI herbicides but not to other groups of AHAS inhibitors, is described in the AHAS1 protein of EMS-mutagenized chickpea line M2033.

## 1. Introduction

Chickpea (*Cicer arietinum* L.), a diploid and self-fertile species, is an important pulse crop worldwide. Chickpea weed management depends mainly on pre-emergence treatments. Several pre-emergent herbicides that inhibit photosynthesis, carotenoid biosynthesis, or the enzymes acetyl coA carboxylase (ACCase) and polyphenol oxidase (PPO) are applied on chickpea [1]. However, the repertoire of postemergent herbicides that are registered for use in chickpea fields is very limited [1,2,3]. A possible solution for this limitation is the selection of chickpea cultivars that are resistant to herbicides such as acetohydroxyacid synthase (AHAS) inhibitors by induced mutations [4].

AHAS, also known as acetolactate synthase (AHAS; EC 2.2.1.6) [5,6,7,8], is the key enzyme in the biosynthesis pathways of branched-chain amino acids (valine, leucine and isoleucine) in plants, fungi and bacteria [9,10]. AHAS is a target for five commercially important herbicide groups: sulfonylureas (SU), imidazolinones (IMI), pyrimidinylthiobenzoates (PTB), sulfonylaminocarbonyltriazolinones (SACN) and triazolopyrimidines (TP) [11]. Inhibition of this enzyme leads to leucine, isoleucine and valine deficiency and results in plant death [6,12,13]. AHAS-inhibiting herbicides are characterized by a broad spectrum of weed control, low toxicity to mammals, high selectivity and high activity, enabling low application rates [7,12,14,15,16]. These advantages have led to the worldwide registration and utilization of more than 30 commercial IMI and SU herbicides since their introduction in the 1980s. These two groups are an important part of the multibillion dollar herbicide market [13]. Both SU and IMI herbicides also show effective broomrape control [17,18,19,20,21,22,23]. In recent years, more and more chickpea fields in Israel and the Middle East have been exposed to two parasitic plants: Egyptian broomrape (*Phelipanche aegyptiaca* Pers.) and crenate broomrape (*Orobanche crenata* Forsk.), due to a change in the growing season in the Middle East from summer sowing (Mar–Apr) to winter sowing (Dec–Feb) [24,25,26]. In Turkey, the IMI herbicide imazethapyr is registered as a pre-emergent herbicide for broadleaf weed control in chickpea [27]. However, this, as well as other IMI herbicides reduce chickpea yield when applied postemergence [28,29]. To be able to utilize AHAS inhibitors to control weeds and broomrape in chickpea fields, a chickpea line that is resistant to at least one or several AHAS inhibitors is required.

The wide use of AHAS-inhibiting herbicides has resulted in the appearance of AHAS-herbicide-resistant weed populations [7,30]. Resistant lines have also been obtained by mutagenesis in *Arabidopsis thaliana* L. [31], sugar beet (*Beta vulgaris* L.) [32,33], canola (*Brassica napus* L.) [34], soybean (*Glycine max* L.) [35], tobacco (*Nicotiana tabacum* L.) [36], cotton (*Gossypium hirsutum* L.) [37], rice (*Oryza sativa* L.) [38] and cereals [39,40]. Some of these mutations confer cross-resistance to several AHAS inhibitors belonging to similar or different chemical groups [10,41]. In most cases of both resistant weeds and crops, resistance is associated with mutation of the AHAS gene. These mutations are caused by substitution of a single highly conserved amino acid residues in the herbicide-binding site of the AHAS catalytic subunit [10,42,43]. Here we report the isolation and characterization of an ethyl methanesulfonate (EMS)-mutagenized F01 chickpea line, M2033, which demonstrates considerable tolerance to IMI herbicides.

## 2. Results

### 2.1. Cross-Resistance of Line M2033 to AHAS Inhibitors

M2033 was found after screening about 3500 M_2_ EMS mutant families for resistance to imazamox. In the screening, the M2033 family survived without any visible damage symptoms, while the wild type (WT) and other tested families had severe symptoms of injury. Except for AHAS resistance, M2033 was phenotypically similar to WT F01 plants.

**Figure 1:**
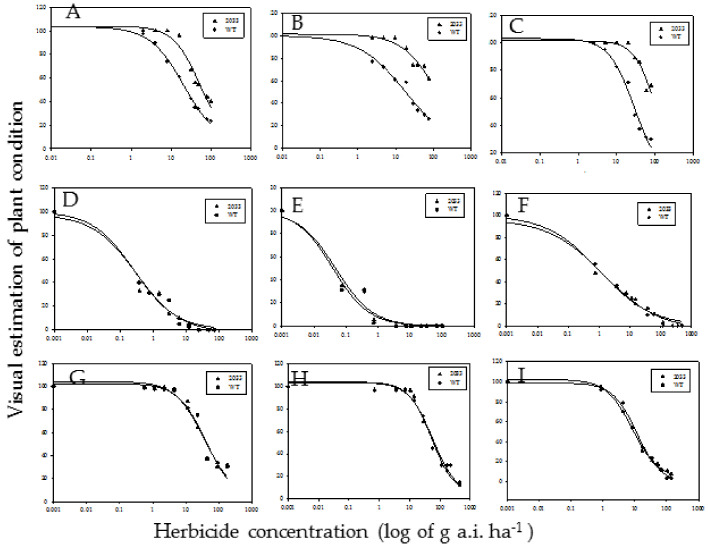
Cross-resistance to AHAS inhibitors. Plant condition was estimated visually 21 days after treatment according to a scale from 100 (healthy plants in nontreated control) to 0 (plant dead). Data were computed by nonlinear regression using Sigma-Plot version 11.01 (SPSS Inc., USA), y = y_0_ + $\frac{a}{1+{\left(\frac{x}{x0}\right)}^{b}}$. The upper asymptote (a) was normalized to 100. (**A**) Imazamox,(**B**) imazapic, (**C**) imazapyr, (**D**) trifloxysulfuron, (**E**) chlorsulfuron, (**F**) sulfosulfuron, (**G**) foramsulfuron, (**H**) propoxycarbazone-sodium, (**I**) pyrithiobac-sodium1.

Resistance of M2033 to various groups of AHAS-inhibiting herbicides was tested. As shown in Figure 1, M2033 was significantly more resistant than WT chickpea plants to all IMI herbicides tested (Figure 1A–C, Table 1), but not to other AHAS inhibitors (Figure 1D–I, Table 1). Under greenhouse conditions, M2033 was significantly more resistant than the WT to up to 100 g a.i. ha^−1^ imazamox (Figure 1A), up to 77 g a.i. ha^−1^ imazapic (Figure 1B), and up to 80 g a.i. ha^−1^ imazapyr (Figure 1C). In the WT plants, damage symptoms were already observed at 4.8 g a.i. ha^−1^ for imazamox (Figure 1A), 2.4 g a.i. ha^−1^ for imazapic (Figure 1B) and 5 g a.i. ha^−1^ for imazapyr (Figure 1C). The damage increased with increasing herbicide rate. The GR_50_ (herbicide dose causing 50% growth reduction) on M2033 for imazamox, imazapic and imazapyr was 53.46 g a.i. ha^−1^ (compared to 21.04 g a.i. ha^−1^ for the WT), 121.24 g a.i. ha^−1^ (18.50 g a.i. ha^−1^ for the WT) and 109.62 g a.i. ha^−1^ (29.11 g a.i. ha^−1^ for WT), respectively (Figure 1A–C). Sensitivity of M2033 to SU herbicides was similar to that of the WT. Both lines were damaged by the lowest rates of these herbicides (Figure 1D–G). The GR_50_ was 0.27 and 0.20 g a.i. ha^−1^ for trifloxysulfuron (Figure 1D), 0.05 and 0.05 g a.i. ha^−1^ for chlorsulfuron (Figure 1E),1.77 and 1.40 g a.i. ha^−1^ for sulfosulfuron (Figure 1F) and 40.23 and 36.66 for foramsulfuron (Figure 1G), for WT and M2033, respectively. Chickpea was less sensitive to propoxycarbazone-sodium than to most SU herbicides (Figure 1H); the GR_50_ values for WT and M2033 were 57.25 g a.i. ha^−1^ and 52.46 g a.i. ha^−1^, respectively. Similarly, there were no differences in sensitivity of the WT and M2033 to pyrithiobac-sodium (Figure 1I); the GR_50_ values for the WT and M2033 were 11.42 g a.i. ha^−1^ and 8.42 g a.i. ha^−1^, respectively.

### 2.2. Line M2033 Contains a Novel Mutation in the AHAS1 Gene

**Figure 2:**
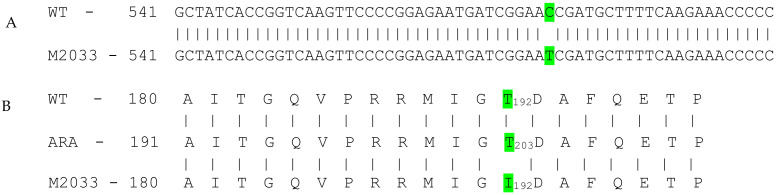
(**A**) WT (line F01) and M2033 chickpea *AHAS1* nucleotides 541–600. The C to T transition at position 578 is highlighted in green. (**B**) WT and M2033 chickpea AHAS1 amino acids 180–199 (191–210 according to *Arabidopsis thaliana* [ARA]). The T to I transition at position 192 (203 according to *Arabidopsis*) is highlighted in green.

DNA sequence analysis of the two *AHAS* genes in line M2033 revealed a single C to T nucleotide transition at position 578 in *AHAS1* (Figure 2A). No other mutations were found in either *AHAS1* or *AHAS2* (data not shown). This mutation caused a highly conserved threonine (T) to isoleucine (I) amino acid substitution at position 192 (203 according to *Arabidopsis*) (Figure 2B).

### 2.3. The Novel Mutation Confers AHAS Resistance in Transgenic Tobacco Plants

**Figure 3:**
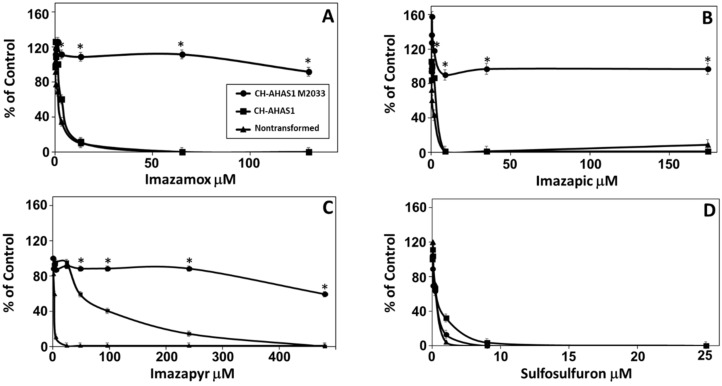
Effect of imazamox (**A**), imazapic (**B**), imazapyr (**C**) and sulfosulfuron (**D**) on root length of 10-day-old nontransformed and transgenic tobacco seedlings expressing the chickpea WT (CH-AHA1) or IMI-resistant (CH-AHA1-M2033) *AHAS1* gene. Each point represents mean ± SEM of five seedlings. * indicate significant difference at *p* < 0.05.

To prove that the novel mutation found in chickpea *AHAS1* leads to IMI resistance, WT and mutated chickpea *AHAS1* under control of the cauliflower mosaic virus (CaMV) 35S promoter was introduced into transgenic tobacco plants. Nontransformed tobacco and homozygous T_2_ seeds of both constructs were germinated on sterile filter paper containing different levels of IMI (imazamox, imazapic, imazapyr) and SU (sulfosulfuron) herbicides. WT tobacco plants were extremely sensitive to all herbicides (Figure 3). Transgenic tobacco harboring the mutant *AHAS1* (CH-AHAS1-M2033) was resistant to all three IMI herbicides (Figure 3A–C), but sensitive to sulfosulfuron (Figure 3D). Transgenic tobacco harboring the WT *AHAS1* (CH-AHAS1) was sensitive to both imazamox and imazapic (Figure 3A,B) and sulfosulfuron (Figure 3D), and showed partial resistance to imazapyr (Figure 3C).

### 2.4. Mutation Inheritance

To determine the inheritance of the mutation, we used 99 chickpea seedlings (10 homozygous for M2033, 14 homozygous WT [IMI-sensitive] and 75 F_2_ population seedlings derived from a cross and self between these two). Sequencing of *AHAS1* in F_2_ plants resulted in 23, 20 and 32 seedlings that were homozygous for the IMI-resistance mutation, WT, and heterozygous, respectively.

**Figure 4:**
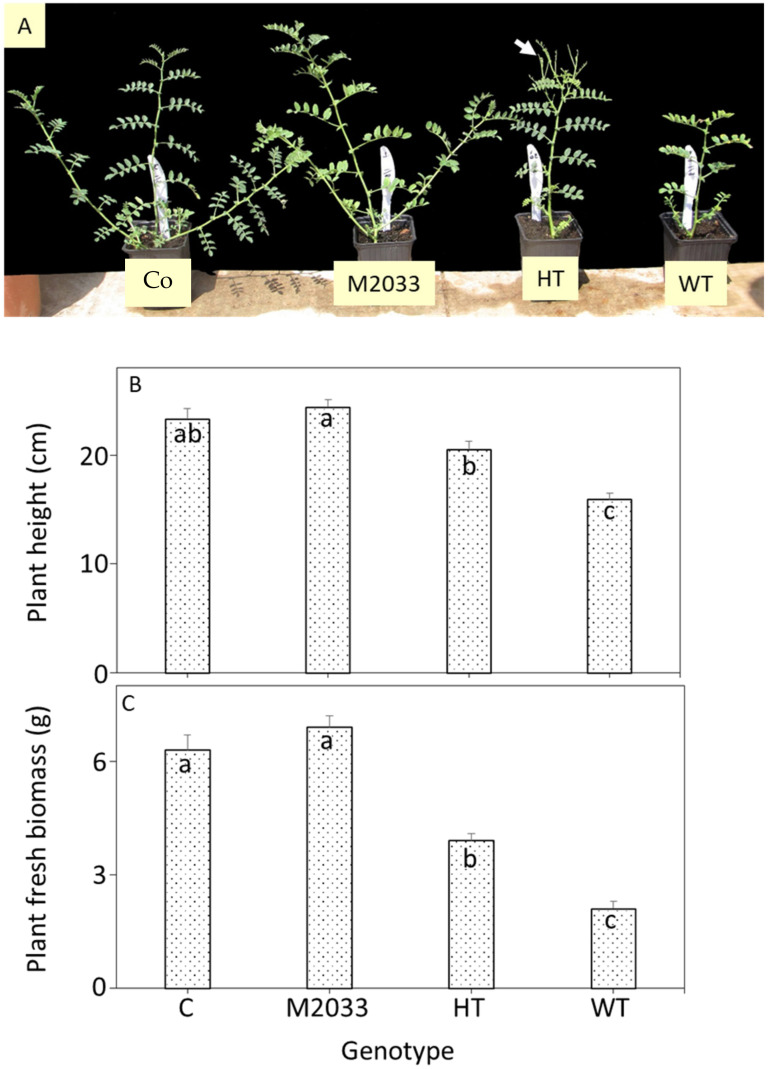
Effect of imazamox treatment (48 g a.i ha^−1^) on chickpea plant phenotype. Co—untreated plants, M2033—homozygous for IMI resistance, WT—homozygous for WT (sensitive), HT—heterozygous plants. (**A**) View of the treated plants. White arrow indicates new branches with smaller leaflets. (**B**) Plant height and (**C**) plant fresh biomass. Bars represent average mean ± SEM of at least 10 plants. Different letters indicate significant differences at *p* < 0.05.

After 2 weeks, 91 (seven homozygous for the mutation, nine homozygous WT and 75 of the F_2_ population) seedlings were sprayed with 48 g a.i. ha^−1^ imazamox. Three weeks later, most heterozygous seedlings had an intermediate phenotype of slower growth rate and increased branching with production of smaller leaflets (Figure 4A), which resulted in reduced plant height (Figure 4B) and biomass (Figure 4C) compared to homozygous M2033 plants.

### 2.5. Mutation Changes AHAS1 Structure

**Figure 5:**
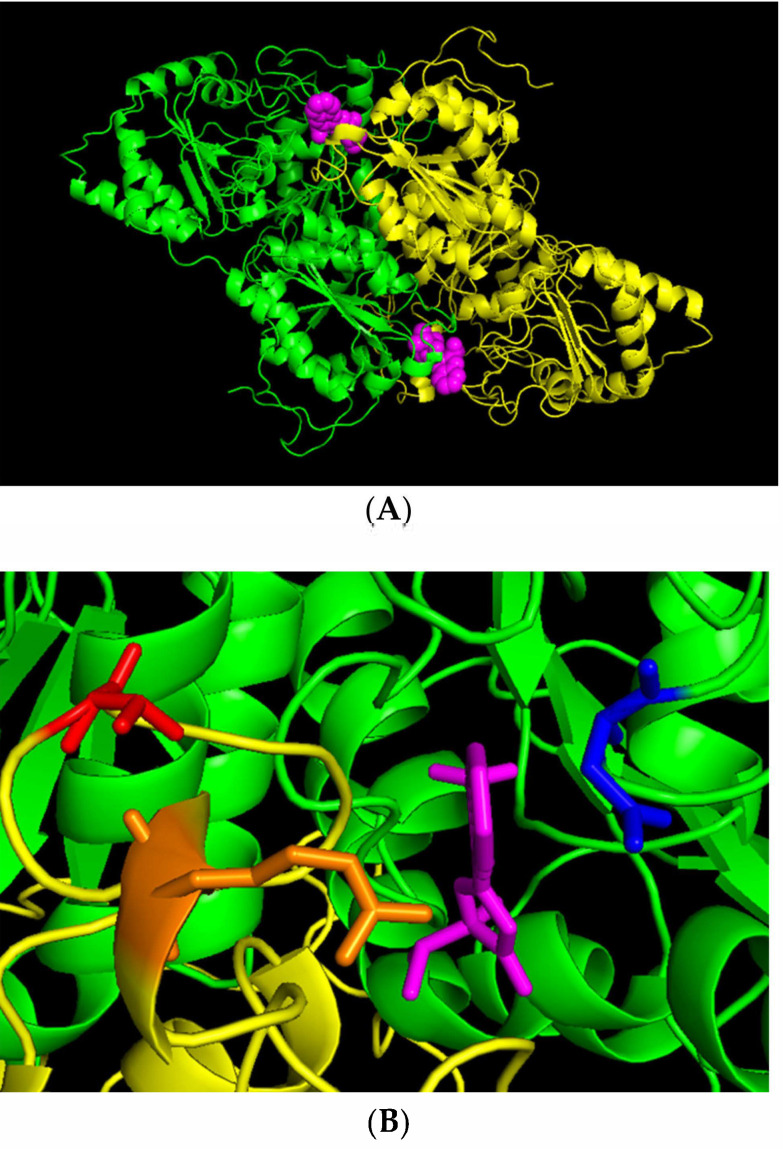
Protein–ligand complex of AHAS1 from *Arabidopsis* with imazaquin (IQ; PDB entry1Z8N). (**A**) Two molecules of IQ block the active channels in the AHAS dimer. (**B**) IQ molecule binding by amino acid residues of the enzyme. Purple—IQ, blue—N654, red—T203, orange—R199.

The crystal structure of *Arabidopsis* AHAS1 in complex with the IMI imazaquin (IQ) [13] revealed that IQ blocks the active channel of the enzyme formed by the interface of two catalytic monomers (Figure 5A). Ligand–protein contact analysis [44] showed that the IQ molecule is bound mainly by N654 (blue, nearest distance to IQ is 2.9 A), T203 (red, nearest distance to R199 is 3.4 A, probably forming a weak H-bond with the backbone oxygen of R199), and R199 (orange, nearest distance to IQ is 3.1 A) (Figure 5B). Replacement of the hydrophilic T203 by the larger hydrophobic I residue has a double effect: steric overlapping and hydrophobic–hydrophilic repulsion with R199, which is expected to strongly affect the IQ-binding pocket.

**Figure 6:**
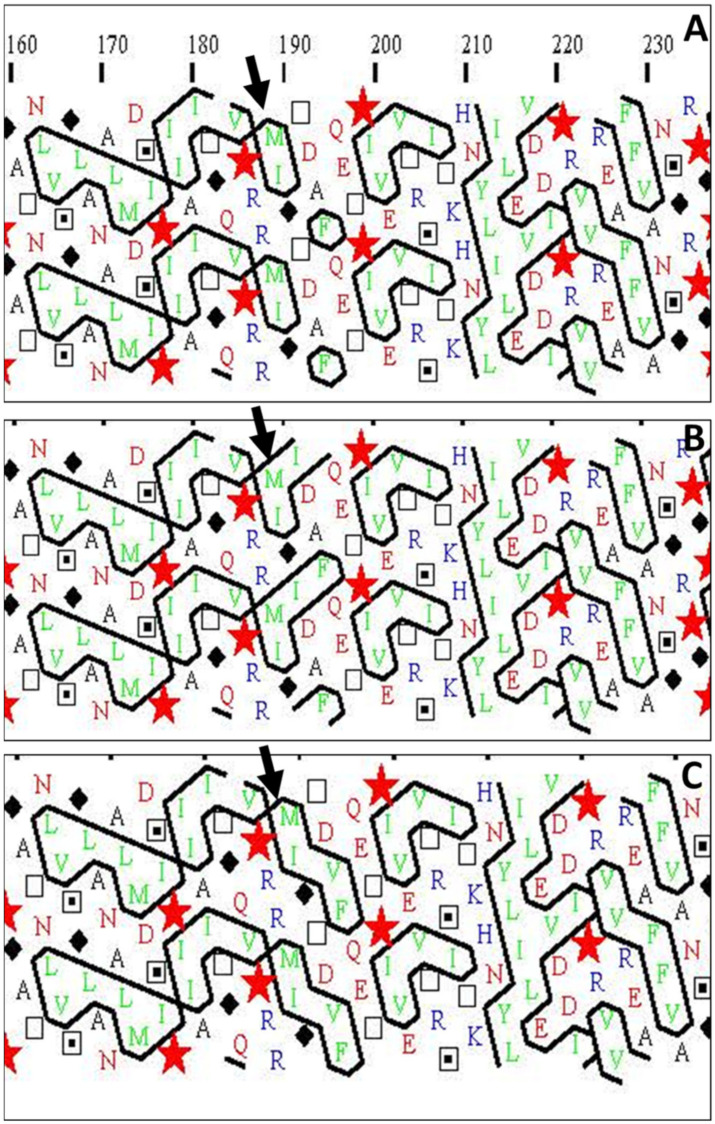
Partial hydrophobic cluster analysis output from (**A**) WT chickpea CH-AHAS1 protein, (**B**) chickpea IMI-resistance mutant (T203V) CH-AHAS1-M2033 protein, and (**C**) chickpea IMI-resistant mutant (A205V) protein [45,46]. Hydrophobic amino acids are in green and hydrophobic clusters are outlined in black. Arrows indicate the difference in the hydrophobic clusters due to a single amino acid change between the three proteins.

Hydrophobic cluster analysis of the WT IMI-sensitive CH-AHAS1 and two mutants—IMI-resistant (T203V) CH-AHAS-M2033 (this work) and the A205V mutant [45,46]—showed that both IMI-resistant mutants change the hydrophobic clusters of the AHAS1 protein (Figure 6), altering their distribution, and leading to changes in the IMI-binding site.

## 3. Discussion

Using EMS mutagenesis, we developed chickpea mutant M2033 resistant to IMI herbicides (Figure S1). In many cases, mutations conferring resistance to one AHAS inhibitor also show cross-resistance to other AHAS inhibitors from different chemical groups [10,30].Therefore, M2033 tolerance to other AHAS inhibitors was evaluated. The mutant tolerated high rates of the IMI herbicides imazamox, imazapic and imazapyr, but did not differ from the WT in sensitivity to SU, propoxycarbazone-sodium or pyrithiobac-sodium (Figure 1).

DNA sequence analysis of the two *AHAS* genes in M2033 revealed a single C to T nucleotide transition at position 578 resulting in substitution of a highly conserved T to I at position 192 (203 according to *Arabidopsis*) (Figure 2). The test with transgenic tobacco plants proved that this mutation confers resistance in the mutant. Three-dimensional structure analyses of the catalytic subunit revealed that IMI herbicides block the channel leading to the catalytic site, thereby preventing AHAS’s true substrate from reaching this site (Figure 5), as previously shown by Duggleby et al. [10] in *Arabidopsis*. T192 (203 according to *Arabidopsis*) is located within a highly conserved region in which other mutations conferring resistance to AHAS inhibitors in other plants have been found [10]. T203, which is located in the coil between two α-helixes, through interaction with A205 and I206, is involved in the formation of the dimer interface. Moreover, ligand–protein contact analysis [44] suggested that the T203I substitution may result in a conformational change in the protein or dimer interface due to the presence of the larger hydrophobic I residue, thus modifying the herbicide-binding site (Figure 5 and Figure 6). Similar results have been obtained with the A205V substitution [45,46,47].

Mutations of at least 17 specific amino acid residues have been identified in bacteria, fungi, and plants as providing resistance to AHAS-inhibiting herbicides. To the best of our knowledge, this is the first report of resistance to AHAS inhibitors via mutation T203I in bacteria, fungi, yeast or plants. Other amino acids in this protein area for which substitution resulted in AHAS-inhibitor resistance are P197, R199 and A205 [10,30]. Of these, mutation A205V leads to cross-tolerance to different groups of AHAS inhibitors [10], in contrast to T203I, which provides tolerance exclusively to IMI herbicides. It is interesting that in chickpea, the single amino acid substitution A194V (A205V according to *Arabidopsis*) in *AHAS1* resulted in a high level of resistance to IMI but not other groups of herbicides [45,46]. It has been shown that the SU- and IMI-binding sites in plant AHAS partially overlap, and share 10 amino acid residues [13]. Six additional residues interact only with the SU-binding site and two bind IMI only. Mutations of A122, P197, A205, or D376 have been suggested to provide cross-tolerance to two or more different groups of AHAS inhibitors [13].

AHAS resistance has been found to show single-gene semidominant [32,35,48] or dominant [49,50] inheritance. In our study, chi-square test gave a ratio of 1:2:1 (χ^2^ = 2.54; *p* = 0.28), indicating a single-gene semidominant inheritance model. Similar results of semidominant inheritance have been obtained in IMI-resistant chickpea lines with mutation A194V (position 205 according to *Arabidopsis*) in *AHAS1* [45].

The IMI-resistant M2033 chickpea mutant may be successfully introduced in agricultural practice, allowing postemergence application of IMI herbicides to control weeds and broomrape in chickpea fields.

## 4. Materials and Methods

### 4.1. Mutagenesis and Characterization of M2033 Mutant

#### 4.1.1. Mutagenesis

WT F01 chickpea breeding line seeds were used for mutagenesis. In order to determine the EMS concentration for the mutagenesis, a preliminary experiment, a multiple dose-response curve of the effect of EMS on chickpea seeds germination was performed (data not shown). To produce M_1_ seeds, approximately 7000 WT F01 M_0_ seeds were allowed to swell in water for 10 h and then exposed to the mutation inducer EMS at a concentration of 4% (*vol*/*vol*) which, according to the dose-response curve, decreases seed germination by 50%. After shaking at 50 rpm for 10 h, the EMS was removed, and the seeds were washed under running tap water for 14 h. The M_1_ seeds were dried under airflow for 48 h and delivered to Shorashim Nursery Ltd., Israel, to produce seedlings. The seedlings were planted, grown and left to self-pollinate in a field at the Western Galilee experimental farm, Israel (32°55′ N 35°04′ E) to produce M_2_ seeds.

#### 4.1.2. Screening for Imazamox Resistance

About 3500 mutant families (each derived from a single M_1_ plant) were used to screen for imazamox resistance. Eight seeds from each family were sown in standard 128 styrofoam growing trays. One-month-old seedlings were sprayed with imazamox at 144 g a.i. ha^−1^, which is twice the recommended rate for weed control in faba bean. Imazamox was applied with a backpack sprayer delivering 200 L ha^−1^ at 300 kPa through T-Jet 11,015 nozzles (Echo SHR210, Echo Interactive Ltd., Israel). Imazamox-treated and non-treated WT seedlings were used as positive and negative controls, respectively. Visual evaluation of plant damage was conducted 3 weeks after treatment, the time required for maximal damage caused by AHAS-inhibiting herbicides. The observation revealed survival of all eight plants belonging to family M2033 with no visible damage symptoms, while the WT and other tested families had severe symptoms of injury. Each of resistant plants were transferred separately to 3 L pots and left to self-pollinate to produce M_3_ seeds. Self M_3_ seeds were used to produce homozygous M_4_ seeds that were used for further analysis.

#### 4.1.3. DNA Extraction and PCR Amplification

Total genomic DNA was extracted from young leaves of 2-week-old M2033 and WT M_4_ plants homozygous for imazamox resistance as previously described by Fulton et al., 1995 [51]. Primer design was done using DNAMAN 4.2 software, PCR amplification, electrophoresis in 1.5% agarose gel and sequence analysis of the *AHAS* genes were performed as described by Schreiber et al. [52] with several modifications of the PCR reaction: annealing was at 55 °C for 30 s and synthesis was at 72 °C for 60 s. For each of the two chickpea *AHAS* genes, five sets of primers purchased from Syntezza Bioscience Ltd. (Israel) were used (Table 2).

#### 4.1.4. Genotype Determination

**Figure 7:**
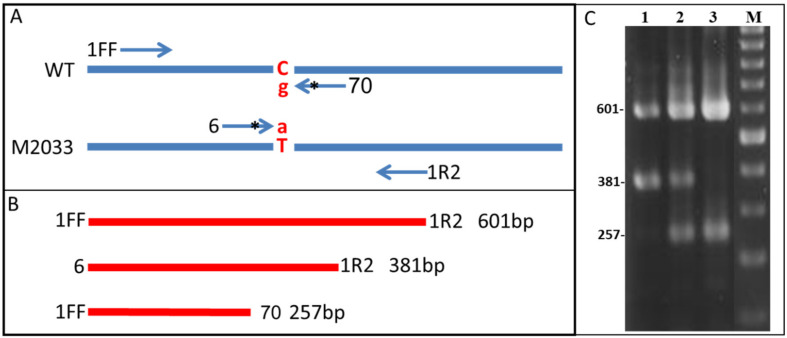
Genotype determination by PCR. (**A**) PCR scheme utilizing two flanking primers: forward primer 1FF and reverse primer 1R2, and two allele-specific primers: forward mutant-specific primer 6 and WT-specific primer 70 (* represents mismatch, and lowercase letters g and a are the third base of these primers, respectively). Uppercase letters C and T are the nucleotides at position 578 of the WT and M2033 chickpea lines, respectively. (**B**) Expected amplified PCR fragments. (**C**) Ethidium bromide-stained representative PCR fragments of the homozygous WT plant (lane 1), heterozygous plant (lane 2), homozygous M2033 plant (lane 3), and 100-bp DNA ladder (lane M). The size of PCR-amplified fragments is shown on the left.

Genotype determination of WT and M2033 chickpea plants utilizing four-primers PCR amplification was performed as previously described by You et al. [53]. This PCR utilized two flanking primers: forward primer CACCACCTCCACTTTCATA (1FF) and reverse primer CTTGGGAAGCCTGGAGAG (1R2), and two allele-specific primers: forward mutant-specific primer CCCGGAGAATGATCGGgAT (6) and WT-specific primer GGTTTCTTGAAAAGCATaGG (70) (small letters represent mismatches) (Figure 7A). This PCR gave rise to amplified products of 601- and 381-bp fragments for homozygous WT plants, 601- and 257bp fragments for homozygous M2033 plants, and 601-, 381- and 257-bp fragments for heterozygous plants (Figure 7B,C).

#### 4.1.5. Determination of Cross-Resistance to AHAS Inhibitors

Chickpea seeds of line F01 and M_4_ homozygous AHAS resistant mutant line M2033 were germinated in 0.5-kg pots containing Newe Ya’ar soil (medium-heavy clay–loam soil containing, on a dry weight basis, 55% clay, 23% silt, 20% sand, 2% organic matter, pH 7.1), one plant per pot, in a net house. Three weeks after sowing, plants were sprayed with increasing rates of (i) the IMI herbicides imazamox (Pulsar^®^, BASF, Zurich, Switzerland)—2, 4, 8, 16, 32, 40, 48, 80 and 100 g a.i. ha^−1^, imazapic (Cadre^®^, Luxembourg Industries Ltd., Tel-Aviv, Israel)—2.4, 4.8, 9.6, 19.2, 28.8, 38.4, 57.6 and 76.8 g a.i. ha^−1^, and imazapyr (Arsenal^®^, BASF, Zurich, Switzerland)—2.4, 4.8, 9.6, 19.2, 28.8, 38.4, 57.6 and 76.8 g a.i. ha^−1^; (ii) SU herbicides trifloxysulfuron (Envoke^®^, Syngenta, Basel, Switzerland)—0.375, 0.75, 1.5, 3, 6, 12, 24, 48 and 72 g a.i. ha^−1^, chlorsulfuron (Glean^®^, FMC, Harboøre, Denmark)—0.075, 0.375, 0.75, 3.75, 7.5, 11.25, 22.5, 33.75, 67.5 and 101.25 g a.i. ha^−1^, sulfosulfuron (Monitor^®^, Adama-Agan, Ashdod, Israel)—0.75, 3.75, 7.5, 11.25, 15, 37.5, 60, 120, 240, 360 and 480 g a.i. ha^−1^, foramsulfuron (Equip^®^, Bayer, Leverkusen Germany)—0.56, 1.125, 2,25, 4.5, 11.25, 22.5, 45, 90 and 180 g a.i. ha^−1^; (iii) SACN herbicide propoxycarbazone-sodium (Olympus^®^, Bayer, Leverkusen Germany,)—0.7, 3.5, 7, 10.5, 14, 28, 56, 112, 168, 224 and 448 g a.i. ha^−1^; (iv) PTB herbicide pyrithiobac-sodium (Staple^®^, KUMIAI, Tokyo, Japan)—0.85, 4.25, 8.5, 17, 34, 51, 68, 102 and 136 g a.i. ha^−1^. Plants sprayed with water were used as a control. Plant condition was estimated visually on a daily basis for 21 days according to scale from 100 (healthy plants in nontreated control) to 0 (plant dead). The experiment was conducted with five replicates.

#### 4.1.6. Determination of Mutation Inheritance

To determine the inheritance of the mutation, we grew 99 seedlings (10 homozygous for the IMI-resistance mutation, 14 homozygous WT [IMI-sensitive] and 75 F_2_ population derived from a cross between these two followed by self-pollination) in 200 mL pots in a net house. Crossing was made as previously described by Dahiya, 1974 [54]. DNA was extracted from each seedling for genotype determination as described in Section 4.4. After 2 weeks, 90 (seven homozygous for the mutation, eight homozygous WT and 75 F_2_ population) seedlings were sprayed with 48 g a.i. ha^−1^ imazamox (in 200 L tap water) and nine control seedlings were sprayed with tap water. Three weeks after imazamox treatment, plant height and fresh weight were determined for each genotype.

### 4.2. Confirmation of the Resistance Mechanism on Transgenic Plants

#### 4.2.1. Construction of Chickpea AHAS Genes and Agrobacterium Transformation

**Figure 8:**
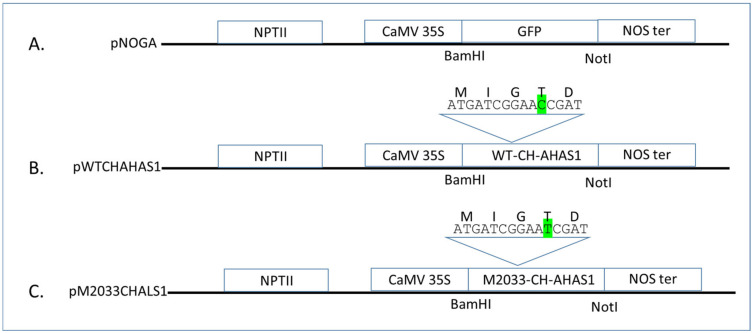
Plasmid constructs used in this study. (**A**) pNOGA—a pBIN19 derivative containing GFP between the CaMV 35S promoter and the NOS terminator (NOS ter) and the neomycin phosphotransferase II (NPTII) gene as the selectable marker. (**B**) pWTCHAHAS1 in pNOGA derivative, in which the native, imazamox-sensitive chickpea *AHAS1* gene was inserted between *Bam*HI and *Not*I, replacing the GFP gene. (**C**) pM2033CHAHAS1 in pNOGA derivative, in which the mutated chickpea *AHAS1* gene, conferring imazamox resistance was inserted between *Bam*HI and *Not*I, replacing the GFP gene. DNA and amino acid sequences near the mutation are indicated. DNA point mutation is shown highlighted in green.

Both WT and mutated (M2033) chickpea *AHAS1* genes were synthetically synthesized (Hylab laboratory, Israel) and 2002 bp DNA fragment was subcloned into *Bam*HI–*Not*I sites of pNOGA, a pBIN19 derivative containing a green fluorescent protein (GFP) (Figure 8A). This gave rise to pWTCHAHAS1 and pM2033CHAHAS1 containing WT and mutated (M2033) *Cicer arietinum AHAS1* genes between the CaMV 35S promoter and the nopaline synthase (NOS) terminator replacing the GFP gene, respectively (Figure 8B,C). Both pWTCHAHAS1 and pM2033CHAHAS1 plasmids were electrotransformed into *Agrobacterium tumefaciens* EHA101. For *Nicotiana tabacum* transformation, *Agrobacterium* harboring the pWTCHAHAS1 and pM2033CHAHAS1 plasmids were grown in 20 mL LB medium containing 100 mg mL^−1^ kanamycin and 100 µM acetosyringone at 26–28 °C overnight in the dark with continuous shaking at 200 rpm. A 2-mL aliquot of the overnight *Agrobacterium* culture was diluted in 18 mL of the same medium and grown for an additional 4–5 h to an optical density at 600 nm (OD_600_) of 0.5–1.0. Then the bacterial culture was centrifuged for 10 min at 160× *g*, room temperature, and finally diluted to OD_600_ = 0.5 with liquid Murashige and Skoog (MS).

#### 4.2.2. Plant Transformation

For *N. tabacum* transformation, healthy, young, undamaged leaves were collected from sterile plants. Leaves were immersed in 4–5 mL of liquid Murashige and Skoog mediuim (MS) in a petri dish (to prevent drying), cut into small squares and placed upside down in induction medium (MS with 1 mg L^−1^ benzyladenine (BA) and 2 mg L^−1^ naphthaleneacetic acid (NAA) +100 µM acetosyringone) for 24 h at 25–26 °C in the dark. Then explants were immersed in *Agrobacterium* culture for about 1–2 min, transferred for 1 min onto a sterile Whatman paper for drying, and then returned to the previous plates and incubated in the dark for 2–3 days for co-cultivation. After co-cultivation, the explants were transferred to selection medium (MS plus 1 mg L^−1^ BA, 0.1 mg L^−1^ NAA, 500 mg L^−1^ claforan [cefotaxime sodium] and 200 mg L^−1^ kanamycin). These explants were transferred to the same medium every 10–14 days. Calli appeared after 2 weeks and small plantlets appeared after about 4 weeks. These plants were transferred for elongation to MS with 0.1 mg L^−1^ BA, 500 mg L^−1^ claforan and 200 mg L^−1^ kanamycin. Developed plantlets (2 cm) were transferred into rooting media (MS without hormones plus 500 mg L^−1^ claforan and 200 mg L^−1^ kanamycin, in Magenta pots). Roots appeared after 15–21 days. Rooted plants were transferred to soil for hardening for 10 days and then into pots for seed production.

#### 4.2.3. Examination of IMI Resistance in Transgenic Plants

For the germination test, about 20 *N. tabacum* WT and homozygous T_2_ seeds containing plasmids pWTCHAHAS1 and pM2033CHAHAS1 were placed on top of two layers of Whatman No. 1 filter paper previously wetted with tap water containing 0, 0.05, 0.1, 0.25, 0.5, 1, 2.5, 5, 10, 25, 50, 100 or 250 µM imazamox; 0, 0.48, 0.96, 2.4, 4.8, 9.6, 24, 48 or 240 µM imazapyr; 0.05, 0.1, 0.2, 0.4, 0.9, 1.7. 8.7, 35, or 174 µM imazapic; or 0, 0.025, 0.05, 0.1, 0.2, 1, 4, 20 or 40 µM sulfosulfuron in a petri dish. After 10 days at 25 °C, root length of five representative seedlings was determined.

### 4.3. Mutation Changes AHAS1 Structure

#### 4.3.1. Ligand–Protein Contact Analysis

Ligand–protein contact analysis, which predicts the binding forces obtained after chemically modified ligands, was conducted using LPC software [44].

#### 4.3.2. Hydrophobic Cluster Analysis

Hydrophobic cluster analysis, which considers that hydrophobic amino acids are nonrandomly distributed and tend to form hydrophobic clusters [55], was conducted utilizing a web-based interface at portal.py?form=HCA#forms::HCA.

### 4.4. Statistical Analysis

The results of the experiments on tobacco plants and the influence of the imazamox on chickpea plant phenotype were subjected to ANOVA using JMP Software, version 5.0 (SAS Institute Inc., NC, USA). Data were compared by LSD on the basis of the Tukey–Kramer honestly significant difference test (*p* < 0.05) and by standard errors of the means (SEM). Nonlinear regressions, using Sigma-Plot version 11.01 (SPSS), were computed for the chickpea cross-resistance to AHAS inhibitors. The chi-square test was used to determine mutation inheritance.

All experiments were conducted twice; the comparison of the two experiments was performed using Fisher’s *t*-test. Data were combined based on the homogeneity of the variances.

## 5. Conclusions

We described the identification of a novel mutation, T192I (T203I according to *Arabidopsis*), in the AHAS1 protein of EMS-mutagenized herbicide-resistant chickpea line M2033. This mutation is located in a highly conserved region of the AHAS protein, in the binding site for IMI herbicides. The mutation was inherited in a single-gene semidominant pattern and confers resistance to IMI herbicides only.

## Figures and Tables

**Table 1:** Nonlinear four-parameter logistic regression y = y_0_ + $\frac{a}{1+{\left(\frac{x}{x0}\right)}^{b}}$ coefficients for comparing chickpea lines sensitivity (visual estimation of plant condition) to AHAS herbicides.

Herbicide	Chickpea Line	R^2^	p	b	x_0_	y_0_
A. Imazamox	WT	0.99	<0.0001	1.02	21.04	3.99
	M2033	0.98	<0.0001	1.30	53.60	3.54
B. Imazapic	WT	0.99	<0.0001	0.70	18.50	0.1
	M2033	0.97	<0.0001	0.95	121.24	1.54
C. Imazapyr	WT	0.99	<0.0001	1.31	29.11	3.1
	M2033	0.95	<0.0001	1.57	109.62	1.47
D. Trifloxysulfuron	WT	0.99	<0.0001	0.69	0.27	0.00
	M2033	0.99	<0.0001	0.65	0.20	0.00
E. Chlorsulfuron	WT	0.96	<0.0001	0.77	0.05	0.00
	M2033	0.98	<0.0001	0.75	0.05	0.00
F. Sulfosulfuron	WT	0.99	<0.0001	0.58	1.77	3.3
	M2033	0.99	<0.0001	0.48	1.40	3.6
G. Foramsulfuron	WT	0.98	<0.0001	1.03	40.23	2.33
	M2033	0.98	<0.0001	0.98	36.33	4.16
H. Propoxycar-bazone sodium	WT	0.99	<0.0001	1.11	57.25	3.42
	M2033	0.99	<0.0001	1.16	52.46	3.88
I. Pyrithiobac sodium	WT	0.99	<0.0001	1.15	11.42	1.03
	M2033	0.99	<0.0001	1.06	8.42	2.57

**Table 2:** Primer sets used in this study for the determination of the two chickpea *AHAS* genes.

Primer Set	Gene	Forward Primer (5′→3′)	Reverse Primer (5′→3′)	Product Size (bp)	Amplified Region
1	1	TAAACTCGAACTCCATCATTCA	GAATATACCGCCTTGTTCGTGA	443	0–414
2	1	CCACCGCACCATCCTCCATAAC	AACGCAAACTTCATGTCAGCAC	582	173–755
3	1	TCCTAGGGTTGTTAGAGAGGCTTT	AACGCAAACTTCATGTCAGCAC	564	659–1223
4	1	TGATTCGGCTGAAATTGGGA	TCAACAACAACAGCATCAGGG	413	1155–1568
5	1	GACAATGGTTAACTTCGGGTGGAC	CTGCCCAATCAATCAGTAACTCC	521	1471–1992
6	2	AAACAATAGAGATTTTAAAGGCC	TTCGAGGTTCGTCAAGGGCA	250	0–250
7	2	ACTCCCCTCCCCTCAACCGAACAA	TTCTAAATGTGACTCAGAAGGTGA	636	192–828
8	2	GGCTAGGTTACCAAAGTCACCTTC	TCCTATTAATCCCCCTCAAAGCC	443	788–1231
9	2	GTGTCAGTTTGTGGGGATTTA	CTCTGTTGTGGTTGACATCGA	383	1182–1565
10	2	ATGTGGTCTGCTCAATTTTATAGT	ACATAATCGGCATCAAGATAAACC	582	1425–2007

## Data Availability

Date is contained within the article and Supplementary Materials.

## Supplementary Materials

The following are available online at https://www.mdpi.com/article/10.3390/plants10122791/s1. Figure S1. Influence of imazamox on chickpea plants. (A) WT (F01), and (B)—M2033. Herbicide rates in g a.i. ha-1 are indicated on the pots. The picture was taken 4 weeks after treatment.
