# Supplementary material for: Novel Mutation in the Acetohydroxyacid Synthase (AHAS), Gene Confers Imidazolinone Resistance in Chickpea Cicer arietinum L. Plants
Source: Plants (Basel). 2021 Dec 16;10(12):2791. doi: 10.3390/plants10122791 (PMC8704328; doi:10.3390/plants10122791)
Supplement: Supplementary file 1 [file plants-10-02791-s001.zip › plants-1437507-supplementary.pdf]

6. Supplementary Materials

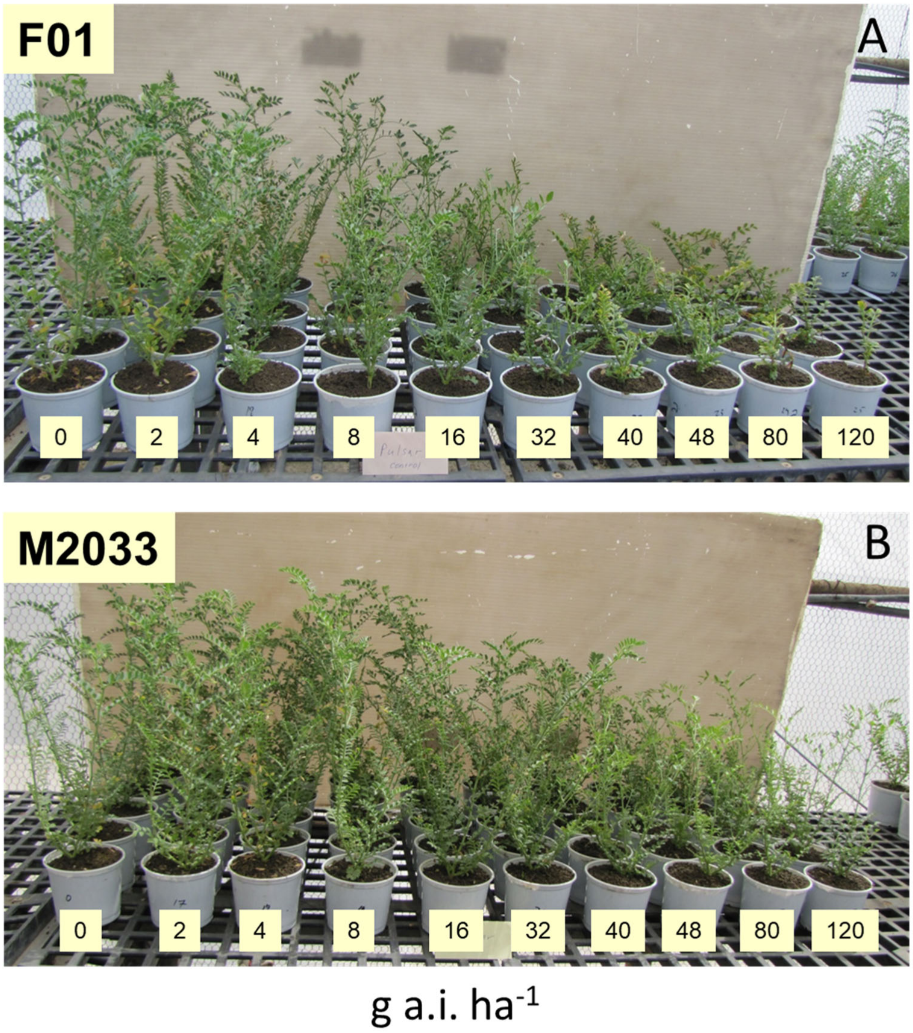

Figure S1. Influence of imazamox on chickpea plants. (**A**) WT (F01), and (**B**) - M2033. Herbicide rates in g a.i. ha^{-1} are indicated on the pots. The picture was taken 4 weeks after treatment.
